# Enhancing mobile EEG: Software development and performance insights of the DreamMachine

**DOI:** 10.1016/j.ohx.2025.e00689

**Published:** 2025-08-19

**Authors:** Paria Samimisabet, Laura Krieger, Marc Vidal De Palol, Deniz Gün, Gordon Pipa

**Affiliations:** Institute of Cognitive Science, Osnabrueck University, 49074 Osnabrueck, Germany

**Keywords:** DreamMachine, EEGDroid, Mobile EEG, Electroencephalography (EEG), Android application, Eyes open/closed

## Abstract

Electroencephalography (EEG) is widely used in fields such as neurology, cognitive neuroscience, sleep research, and mental health. It records brain electrical activity to study neurophysiological functions. Numerous EEG and mobile EEG systems are available. However, adherence to the standards set by the International Federation of Clinical Neurophysiology (IFCN) is essential for ensuring high-quality data collection in clinical environments. The DreamMachine, a mobile EEG device, complies fully with these standards, offering 24-channel recordings at 250 Hz, Bluetooth Low Energy (BLE), and capabilities for electrooculography (EOG) and electrocardiography (ECG). Its low cost makes it an accessible option for EEG studies. The software architecture of the open-source DreamMachine is detailed in this study. Focus is placed on data compression and communication between the device and its companion Android application. The details of the Android application’s features, including gain settings, bits per channel, filters, bit-shifting, and safety factors, are investigated. Subsequently, the system’s performance is evaluated through a standard eyes-open/eyes-closed experiment, comparing its results with a laboratory EEG system across a significant number of participants to assess the performance of the DreamMachine system.

## Graphical abstract

**Figure:**
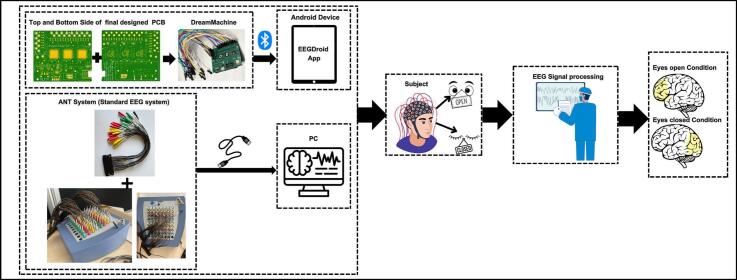

**Specifications table**

**Table:** 

Hardware name	*DreamMachine*
Subject area	• Neuroscience • Educational tools and open-source alternatives to existing infrastructure • General
Hardware type	• Measuring physical properties and in-lab sensors • Electrical engineering and computer science • Neuroscience tools • Other: Mobile EEG device
Closest commercial analog	*OpenBCI, SMARTING mobi*
Open-source license	*GNU General Public License v3.0*
Cost of hardware	*Around 150 Euro*
Source file repository	https://doi.org/10.5281/zenodo.14627406

## 1 Hardware in context

EEG is one of the most non-invasive methods for recording brain activity [1]. A portable version, known as mobile EEG, allows for its use outside of traditional laboratory settings [2]. EEG systems have a wide range of applications, and their versatility has been explored across various domains [3]. Their significance is evident in both scientific research and practical applications. For instance, EEG is frequently employed in cognitive neuroscience, where brainwave patterns are analyzed to reveal the neural mechanisms underlying cognitive processes such as attention, memory, perception, and language, offering valuable insights into the complexities of human cognition [[4], [5], [6]]. In clinical diagnosis and monitoring, EEG assumes a pivotal role in diagnosing and monitoring neurological disorders like epilepsy, sleep disorders, and brain injuries by recording abnormal electrical activities to facilitate precise diagnoses and tailored treatment plans, even assisting in pinpointing abnormal brain activity locations for surgical planning in epilepsy cases [[7], [8], [9]]. In neurofeedback therapy, EEG-based techniques are employed in therapeutic settings to enable individuals to regulate brain activity patterns through real-time feedback, offering significant benefits for conditions such as attention deficit hyperactivity disorder (ADHD) and anxiety disorders [10,11]. Furthermore, EEG plays a pivotal role in developing brain computer interfaces (BCI), enabling individuals with severe motor disabilities to control external devices using brain signals, ranging from computer interactions to immersive virtual reality experiences [12,13]. EEG also enhances emotional and mood analysis by revealing how the brain processes emotions in response to stimuli, contributing valuable insights to affective computing and psychological research by uncovering patterns of emotional states [14]. Additionally, EEG is utilized in educational research to study learning processes and interventions, allowing researchers to assess the impact of teaching methods on brain activity and cognitive engagement, ultimately refining instructional strategies [15,16]. In psychological studies, EEG aids in investigating brain processes related to decision-making, problem-solving, and social interactions, providing valuable insights into the neural foundations of human behavior and cognition [[17], [18], [19], [20]]. Notably, many of these applications can yield meaningful conclusions without requiring high resolution or many electrodes. This trend is accelerated by the rise of deep learning and other machine learning approaches, which enhance signal processing and enable reliable conclusions from smaller datasets [21]. Although these tools and algorithms can compensate for weaknesses in the original data, improving systems for recording high-quality data remains crucial.

A wide range of EEG and mobile EEG systems with various configurations are available on the market [[22], [23], [24]]. However, the IFCN has established standards for EEG devices to ensure high-quality and consistent data recording in clinical settings [25]. These specifications are designed to maintain the accuracy, reliability, and usability of EEG recordings. They include a minimum of 24 channels, a sampling rate of at least 200 Hz, a range of filters, a resolution of no less than 12-bit, and the provision of data in a standard file format. The DreamMachine fully meets all these requirements. This device connects to a companion app on an Android device via Bluetooth technology. It features a lightweight design, offers a battery life of up to six hours, and is capable of recording 24 channels at a 250 Hz sampling rate [16]. Additionally, this device can be easily adjusted to capture EOG and ECG signals if needed. Remarkably, the production cost of the device remains below 150 Euros (as of the design period) [16]. Moreover, every aspect of the project, from a detailed explanation of the hardware configuration to the source code of the companion app, is openly accessible and available for unrestricted use and on GitHub repository.

Following the discussion of relevant research on mobile EEG devices and similar projects, the focus shifts to the software components of the mobile EEG systems. In this study, the software details of the DreamMachine project are investigated. This includes an explanation of data compression and decompression techniques, establishing bidirectional communication between the companion application and the EEG device, and the introduction of the filters used within the application. The analysis concludes with a performance evaluation based on a standard eyes-open and eyes-closed experiment, comparing the DreamMachine device with a laboratory EEG system (asalab™ by ANT Neuro) [26]. For a detailed discussion on the hardware aspects, please refer to [16].

## 2 Hardware description

**Fig. 1:**
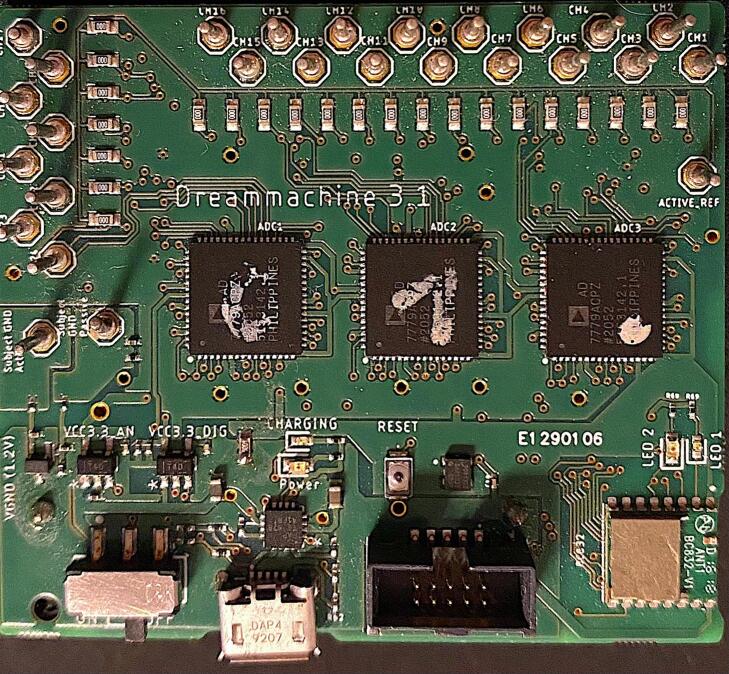
Illustration of the DreamMachine hardware system.

The DreamMachine hardware system is shown in Fig. 1. It is a compact, mobile EEG device that streams real-time brain activity data to a dedicated Android application (EEGDroid) via BLE. The system uses a 24-bit resolution ADC to accurately record neural signals, with configurable options to operate at 10-, 14-, or 16-bit resolution depending on user needs. Users can also select between sampling rates of 250 Hz and 167 Hz.

Physically, DreamMachine is a rectangular board measuring 58.55 × 51.03 mm (L × W), weighing 40 g with the battery and 22 g without. The battery supports up to six hours of continuous recording and weighs approximately 18 g. The system is equipped with 24 EEG channels, corresponding to standard 10–20 electrode locations: FP1, FPZ, FP2, F7, F3, FZ, F4, F8, M1, T7, C3, CZ, C4, T8, M2, P7, P3, PZ, P4, P8, POZ, O1, OZ, and O2. It also includes two additional channels, one for reference and one for ground, for a total of 26 input connections. Each of these 26 channels is accessible via soldered pins on the PCB, enabling users to connect a wide range of electrode types, including stick-on, pads, cap-mounted, wet/gel-based, tattoo, or flexible printed electrodes. This open connector layout supports high flexibility in experimental setups, making the device suitable for diverse applications in research, education, and prototyping.

### 2.1 Data transmission

To transfer data from the DreamMachine system to the Android application, the analog EEG signals must first be converted into digital form using analog-to-digital components. This conversion is performed with 24-bit resolution, ensuring high precision in representing the signals. The system samples data at a rate of 250 Hz, capturing 250 data points per second. However, transmitting raw 24-bit data for each channel would require substantial bandwidth, making the process inefficient and potentially costly. To overcome this limitation, a data compression technique is applied before transmission. This reduces bandwidth usage while preserving signal quality. Without compression, transmitting the full 24-bit data would significantly limit the device’s sampling rate and the number of channels it could support. Instead of sending full 24-bit samples, the data is compressed to 16 bits, which is more practical for wireless transmission. While this reduces some precision, it greatly improves efficiency. The compression method works by transmitting the difference between the current sample and a predicted value rather than the absolute recorded voltage. The prediction is typically based on the previous sample or a weighted combination of earlier samples, using a technique known as difference coding, delta encoding, or Differential Pulse Code Modulation (DPCM) [39]. This approach takes advantage of the strong correlation between successive EEG samples to represent the signal more compactly. Another crucial aspect of the data transmission process is Adaptive Differential Pulse Code Modulation (ADPCM) [40]. ADPCM ensures that only the most relevant 16-bit per channel are transmitted instead of all 24-bit. By encoding the difference between consecutive samples, ADPCM compresses digital signals, maintaining high resolution and incorporating an automatic artifact filter to enhance data quality. These approaches allow the transmission of 24 channels with a sampling rate of up to 250 Hz, preserving high resolution while minimizing the transmitted data volume. Additionally, ADPCM helps filter out artifacts, ensuring higher quality transmitted data. However, some data loss or errors may occur during Bluetooth transmission due to various factors.

It is important to be aware that data loss can occur during Bluetooth transmission, as Bluetooth relies on radio waves for data transfer. Several factors inherent to the nature of wireless communication can contribute to this issue. The physical distance between devices, Bluetooth signal strength, and interference from other wireless signals including Wi-Fi, microwaves, or other Bluetooth devices, can all degrade the quality of the connection. These factors can lead to an unstable Bluetooth connection, which may result in lost or corrupted data packets, causing errors or incomplete data on the receiving device. In addition to environmental factors, device limitations can further exacerbate transmission issues. For instance, high CPU load on the receiving device may hinder its ability to process incoming data efficiently, leading to data loss. Furthermore, outdated Bluetooth hardware or software can make devices incompatible with the latest Bluetooth protocols, which may impact the stability and reliability of the connection. Several considerations should be made to minimize packet loss and avoid decoding errors and signal shifting during Bluetooth transmission.

To optimize Bluetooth transmission and minimize data loss, several strategies should be considered. First, it is essential to ensure that devices are placed within an optimal range, typically 1–10 m, depending on the Bluetooth class, to maintain a strong signal and reduce packet loss. Additionally, positioning devices in areas with minimal physical obstructions, such as walls or metal objects, is crucial to prevent signal attenuation. Next, employing the latest Bluetooth versions, such as Bluetooth 5.0 or later, along with advanced protocols like BLE, can significantly improve transmission reliability, energy efficiency, and resistance to interference. Furthermore, integrating robust error-correction mechanisms, such as Forward Error Correction (FEC) or Automatic Repeat reQuest (ARQ), into the Bluetooth protocol stack can further enhance data integrity and reduce packet loss [41]. Another important consideration is avoiding Radio Frequency (RF) interference, as Bluetooth operates in the 2.4 GHz ISM band, which is shared with devices like Wi-Fi routers and microwaves. To mitigate this interference, using Bluetooth devices that support adaptive frequency hopping (AFH), and dual-band support (2.4 GHz and 5 GHz) can improve connection stability [42]. Reducing the load on the receiving device is also vital; optimizing its CPU performance by closing unnecessary applications or offloading tasks to external hardware can help maintain efficient data processing. Ensuring that both devices have an adequate power supply is equally important, as low battery levels can lead to signal instability and degraded transmission quality. Finally, in environments with multiple Bluetooth devices, such as smart homes or IoT settings, managing the Bluetooth network efficiently, through segmentation or using Bluetooth mesh networks, can help prevent congestion, thereby improving communication reliability and stability over larger areas.

### 2.2 Bluetooth

Bluetooth is the most widely adopted wireless communication method in modern mobile EEG systems, including those with relatively high channel counts and sampling rates. Its popularity stems from several key advantages, including low power consumption, stable connectivity, and a user-friendly pairing process, all of which make it well suited for real-time EEG applications.

The DreamMachine leverages BLE for data transmission, making it especially effective in mobile scenarios such as sleep monitoring, where long battery life and energy efficiency are critical. To operate effectively within Bluetooth’s bandwidth limitations, the system employs a custom compression algorithm that enables efficient transmission of high-resolution EEG data without packet loss or degradation in performance.

While some systems opt for Wi-Fi to support extremely high-throughput needs (e.g., uncompressed, multichannel, high-frequency recordings), Bluetooth remains a reliable and proven solution for most mobile EEG applications. It’s typical range (∼2 m) is generally sufficient when the receiver device, such as a smartphone or tablet, remains near the participant.

In contrast to traditional EEG systems that rely on cabled connections to a computer, which restrict mobility and introduce motion artifacts from cable sway, Bluetooth-based wireless transmission offers a significant improvement. As first demonstrated by Debener et al [43], eliminating cables enhances participant mobility and reduces motion-induced noise, resulting in a more practical and user-friendly experience.

Altogether, Bluetooth, especially when combined with DreamMachine’s efficient compression strategy, strikes an effective balance between efficiency, reliability, and usability, making it a highly suitable and optimized data transmission method for modern mobile EEG systems.

### 2.3 Android application (EEGDroid)

The EEGDroid application, developed as the companion app for the DreamMachine, is built for Android devices using the Java programming language. Its primary functions include receiving and decoding compressed EEG data, real-time signal visualization, recording management, and configuration of the DreamMachine hardware. Users can adjust parameters such as sampling rate, bit resolution, and filter settings directly within the app. EEGDroid also serves as an educational tool, offering basic EEG knowledge and user guidance via its GitHub repository.

To assess performance across devices, the application was tested on two Android tablets, including Samsung Galaxy Tab S4 (Android 10) and Samsung Galaxy Tab S6 (Android 12). In both cases, the app maintained stable BLE connections, displayed real-time EEG data without delay, and reliably saved data files. No compatibility issues or crashes were observed during configuration or recording sessions, demonstrating that EEGDroid performs consistently across different hardware and operating system versions within the tested range.

#### 2.3.1 Gain and bits per channel

The AD7779 component used in the DreamMachine includes a programmable gain amplifier (PGA) with selectable gains of 1, 2, 4, and 8. These gain settings scale the input signal before digitization, effectively increasing the resolution for low-amplitude signals such as EEG. For example, with a reference voltage of 2.5 V, using gain = 1 gives a full input range of ± 2.5 V and a resolution of about 0.298 μ V per LSB. Using gain = 8 reduces the input range to ± 312.5 mV, but increases the resolution to about 0.037 μV per LSB. The output remains 24-bit (16,777,216 codes), but the LSB size changes based on gain. These settings are configurable in the EEGDroid application to allow users to optimize for precision based on signal characteristics.

The term “bits per channel” refers to the number of digital bits used to represent the amplitude of the EEG signal recorded from each channel. In EEG acquisition, analog signals generated by brain activity are sampled and converted to digital values. A higher bit depth allows for finer resolution and a broader dynamic range, meaning smaller voltage differences can be detected.

Within the EEGDroid application, EEG data—originally captured by the AD7779 chip at a high 24-bit resolution—is commonly downsampled to 10, 14, or 16 bits per channel for transmission and storage. This downsampling helps reduce memory usage, bandwidth demands, and processing load. For context, a 10-bit signal can represent values from 0 to 1023, a 14-bit signal from 0 to 16,383, and a 16-bit signal from 0 to 65,535. While higher bit depths preserve more detail and dynamic range, they also generate significantly larger data streams. Therefore, the selected bit depth reflects a balance between the desired signal precision and the limitations of the hardware or software environment.

#### 2.3.2 Filtering

Filtering of EEG signals is a critical step in data preprocessing for experiments. Because EEG signals are low amplitude (in the microvolt range), they are prone to noise contamination from electrical devices, muscle movements, and environmental factors. Filtering is used to eliminate these unwanted components, improving the data’s signal-to-noise ratio (SNR). Specific frequency bands can also be selectively amplified or attenuated to enhance desired EEG components, such as brain waves. Filtering also assists in detecting and removing artifacts like eye blinks, eye movements, and electrode pops, aiding in the accurate interpretation of brain activity. Furthermore, filtering standardizes EEG data across various recording conditions and subjects, facilitating easier comparison of results across studies. For these reasons, DreamMachine offers configurable settings with a range of filtering options, enabling users to select the optimal settings for achieving high-quality EEG signals.

**Fig. 2:**
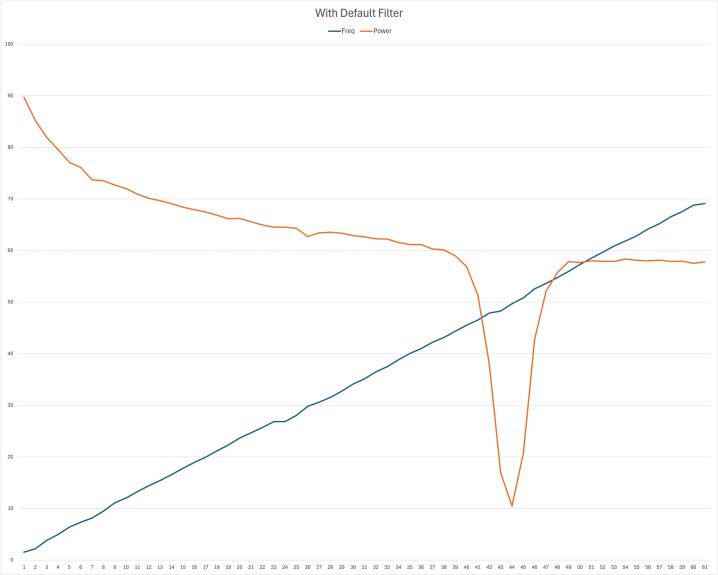

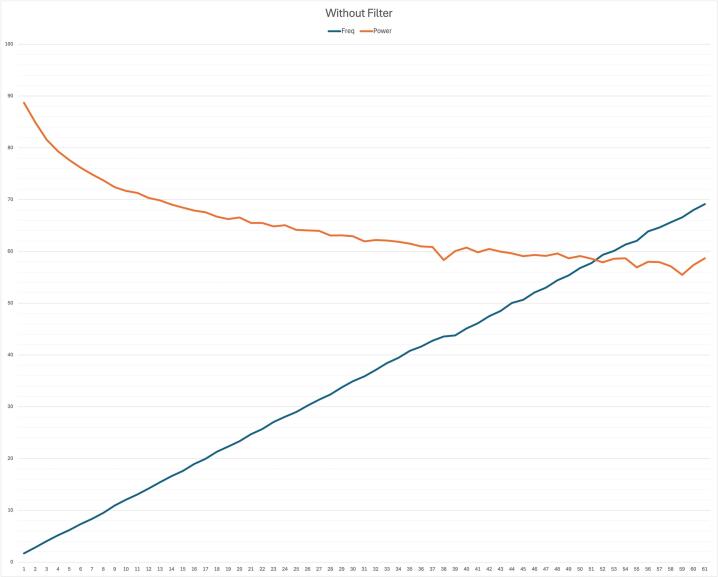
A sweep signal ranging from 1 to 70 Hz is generated using a signal generator and injected into the DreamMachine. The experiment is conducted twice: once with the default filter setting (filter on) and once with all filters disabled (filter off). The blue line represents the sweep signal from 1 to 70 Hz, while the orange line shows the power attenuated at 50 Hz when the filter is active.

Data is initially sampled at a rate of 500 Hz, with filters applied before the data is downsampled to 250 Hz for transmission. The default filters include an infinite impulse response (IIR) filter, a fourth-order high-pass filter with a cutoff frequency of 1 Hz, a fourth-order low-pass filter with a cutoff frequency of 45 Hz, and a band-stop filter with a range of 46-54 Hz. The effect of the default filters is demonstrated in Fig. 2, which shows that the DreamMachine effectively eliminates the 50 Hz noise caused by power line interference.

Other filtering options of the DreamMachine provides are listed in Table 1. Users can customize these settings or disable the filters entirely, depending on the needs of their experiment. While our testing suggests that the default filtering configuration provides optimal performance in most cases, the ideal setup ultimately depends on the specific research question and should be adjusted accordingly.

**Table 1:** Filter options provided by the DreamMachine system.

High-pass Filter	Low-pass Filter	Band-stop Filter
0.8 Hz order 2	45 Hz order 4	46–54 Hz order 6
1.0 Hz order 4	60 Hz order 6	46–54 Hz order 4
1.7 Hz order 4	*−*	48–52 Hz order 6
1.7 Hz order 2	*−*	48–52 Hz order 4
off	*off*	off

#### 2.3.3 Bitshift and safety factor

Even if only the differences between consecutive 24-bit recordings were transmitted, the resulting values would still require full 24-bit representation. This imposes limits on either the sampling rate or the number of channels that can be supported due to bandwidth constraints. To address this, the system tracks a floating average of the signal on the device, and each second, it calculates the standard deviation of the signal. This value is then used to identify the most relevant portion of each 24-bit difference for efficient transmission.

**Fig. 3:**
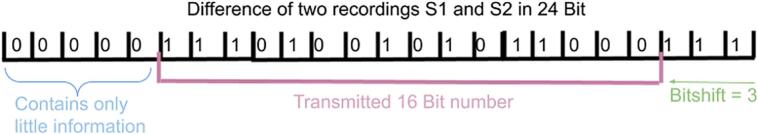
Bitshift Diagram: The calculated difference between S1 and S2 is a smaller value still represented in 24-bit. When the difference is small, the leading bits are zeros and carry no information. In contrast, when the difference is large, the initial bits contain significant information, while the later bits, representing very fine voltages, can be neglected.

Instead of sending the full 24-bit difference, a bit shift value is transmitted alongside the compressed data. This bit shift indicates how many of the most significant bits (from the left) are skipped. As shown in Fig. 3, a larger bit shift enables transmission of larger signal changes but reduces resolution. In contrast, a smaller bit shift provides higher precision but limits the range of representable differences. Each EEG channel determines its optimal bit shift individually based on its recent activity. This bit shift is sent to the companion application, where it is used to reconstruct the signal by applying the shift and adding the result to the previous value.

Advanced users can configure a maximum bit shift or apply a safety factor based on expected signal characteristics. Limiting the maximum bit shift is especially useful in typical EEG recordings (e.g., from healthy participants), where large jumps often indicate artifacts—such as those caused by muscle activity, eye blinks, or electrode disturbances. In such cases, cutting off these peaks does not significantly impact useful data. By setting a maximum bit shift, the system avoids allocating bandwidth to potentially non-informative large changes and instead preserves resolution for subsequent samples.

The safety factor specifies how much greater a single signal change can be relative to the standard deviation measured over the previous second. For example, a safety factor of 8 means that differences up to eight times the recent standard deviation can be transmitted without truncation. To achieve this, four additional high-order bits are used beyond what the algorithm would normally allocate. While this reduces overall resolution, it prevents loss of critical data during sudden large changes. This is particularly relevant in clinical applications such as seizure detection, where large amplitude spikes may carry diagnostic value and should not be discarded as artifacts.

The maximum voltage difference that can be transmitted depends on the number of bits used for encoding the difference, and is calculated using:

$$\Delta max=\frac{2\times Vref}{2^{b}}$$

where VREF = 2.5 V and b is the number of bits used to represent the signal, the minimum detectable voltage change per transmitted step depends on the resolution. For example, with 16-bit resolution, the smallest step is $\frac{2\times Vref}{2^{16}}=76.3\mu V$, and with 24-bit resolution, it is $\frac{2\times Vref}{2^{24}}=298.0nV$. These values represent the smallest voltage differences that can be detected and transmitted, helping to define how much signal detail is preserved or lost under different compression strategies.

#### 2.3.4 Data formats

Facilitating the utilization of our device by numerous researchers and ensuring seamless data management in accordance with established EEG standards has been a paramount consideration. Consequently, we have incorporated two methods for recording and storing data. The first method involves the straightforward saving of recordings directly onto the smartphone or tablet in “CSV” format. Alternatively, particularly during experimental scenarios, data can be streamed through LabStreamingLayer (LSL) [50]. This approach enables impeccable synchronization with triggers and other experimental inputs essential for the processing of EEG data. The LSL format adheres to industry standards, specifically the “xdf” format, and allows for convenient further processing using popular EEG tools such as Fieldtrip and EEGLab [45,46].

### 2.4 Package loss

**Fig. 4:**
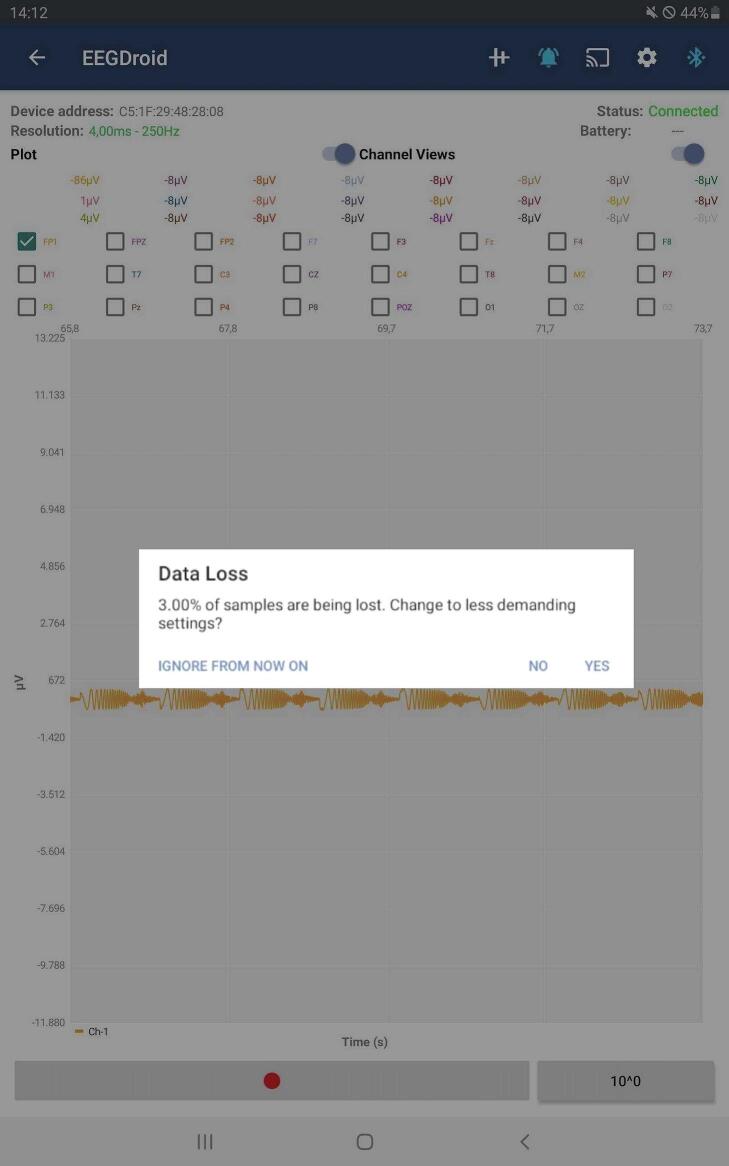
This figure displays the EEGDroid Android application, which is generating a data loss notification. It indicates that 3% of the samples are being lost and suggests changing the current settings to prevent further data loss.

One of the goals of the DreamMachine system is to enable a broad range of people to use the EEG device. Therefore, the device should not only work with powerful end devices with high CPU capabilities and the latest Bluetooth technology but also yield satisfactory results with older devices. However, if sub-optimal settings are selected, data packages can be lost due to a full Bluetooth buffer or an overloaded CPU of the receiving device. As discussed before, the decoding algorithm does not perform optimally if packages are lost since a correlation between subsequent recordings is assumed. This effect is exacerbated if the packages are not evenly sampled and are lost in a set. Therefore, preventing package loss is preferred over maintaining a lower sampling rate, even if the absolute number of transmitted packages might be smaller. To maintain optimal performance, the app actively monitors the occurrence of data package loss and, when necessary, provides recommendations for switching to alternative settings, as depicted in Fig. 4.

### 2.5 State of the art mobile EEG devices

In recent years, there has been a growing interest in using mobile EEG systems across a wide range of experiments. This increased interest is driving the development and innovation of new mobile EEG technologies [44]. A standardized method for evaluating and comparing mobile EEG systems across different studies is essential for selecting the appropriate system for research or developing the next generation of mobile EEG technology. The Categorization of Mobile EEG (CoME) scheme was introduced to address this need. This scheme classifies mobile EEG devices based on several key parameters, including device mobility, participant mobility, system configuration (such as the number of channels, sampling rate, resolution, and other technical features), and contextual factors that may influence the usability and performance of the EEG system. By evaluating these aspects, the CoME scheme aims to enhance the quality and reliability of recorded data and ensure the practical application of mobile EEG technology. In the paper by Anthony Bateson and his colleagues [27], the CoME scores for 15 mobile EEG experiments conducted with five different devices developed over the past twelve years were evaluated, including their own. The average CoME scores for these devices are as follows: 3.5 out of 5 for device mobility (D), 1 out of 5 for participant mobility (P), 9 out of 20 for system specification (S) (with the mean used when ranges were provided), and a channel count (C) of 10.3. In comparison, our device scores 4D, 1P, 10-12S, and 24C. The bit resolution is ambiguous, as we sample at 24-bit resolution (scoring 4) but transmit at 16-bit resolution (scoring 2). Considering this, DreamMachine meets or exceeds the average performance of current mobile EEG devices in the evaluated categories. The following paragraph highlights three recent devices to underscore the unique features of DreamMachine. It demonstrates that DreamMachine is unparalleled in being open-source and cost-effective while maintaining performance competitive with commercial mobile EEG systems.

In comparison to the mobile EEG system presented by Anthony Bateson [27], several distinct approaches and advantages are offered by DreamMachine. The key differences include the use of Wi-Fi instead of Bluetooth, the implementation of the companion application in C# rather than Java, and the unique method of transmitting experimental triggers. In Anthony Bateson’s study, Wi-Fi was used because of its higher bandwidth compared to Bluetooth. However, in DreamMachine, an innovative encoding scheme effectively mitigates Bluetooth’ slower bandwidth and leverages the advantages of BLE. For further details, see Bluetooth section. The decision to implement the application in Java was made due to its widespread use and prevalence. Data indicates that Java is the second most learned programming language after Python [28]. The versatile, open-source application developed in a widely known programming language allows researchers to easily customize the application to suit their experimental needs. An additional advantage of the DreamMachine lies in the seamless storage of triggers. Unlike the device presented in Anthony Bateson’s study, where triggers are sent via a designated channel, DreamMachine is compatible with LabRecorder and capable of streaming data using LSL. This compatibility enables researchers to continue using established methods for conducting experiments. Moreover, in this study, a prototype of the application has been developed to save triggers and experimental cues directly as a CSV file, allowing all channels to be utilized for recording EEG data. Although Bateson’s project incorporates an impedance check within their companion app—a feature that aids in setting up connections and improving recording quality—this functionality is currently absent in the DreamMachine system. However, the inclusion of this valuable feature is planned for a future version of the application.

A widely used and well-established mobile EEG device is the SMARTING mobi3, which has been employed in various research contexts, including auditory attention decoding, saliency detection, visual selective attention tasks, and BCI development [[29], [30], [31]]. The system records 24 channels with sampling rates between 250 and 500 Hz and is compatible with the Lab Streaming Layer (LSL) [32]. The manufacturer also offers an advanced version, SMARTING pro4, which includes additional features such as 32-channel support, sampling rates up to 1000 Hz, 3D motion tracking, automatic artifact removal, and onboard data storage. In contrast to commercially closed systems like SMARTING, the DreamMachine was developed with a different set of goals, particularly focusing on open-source accessibility, cost-effectiveness, and customizability. The DreamMachine’s hardware and software are openly documented, allowing researchers to inspect, adapt, and extend the system to suit their specific experimental or educational needs. This makes it especially suitable for teaching environments, hackathons, and research in resource-limited settings. While SMARTING systems offer advanced technical capabilities and high-performance specifications, a direct performance comparison is beyond the scope of this paper. Instead, DreamMachine aims to serve as a complementary solution, enabling broader access to EEG technology through openness and affordability rather than competing with premium systems on specification alone.

The Emotiv Insight, first released in 2014, offers a range of commercially available EEG brainwear devices [24,33]. Their systems, equipped with between five and 32 sensors, record EEG data at a 128 Hz sampling rate and detect head movements. The systems connect to PCs and mobile devices via Bluetooth. The various devices are priced between $500 and $2100. In addition to the hardware, the company provides an ecosystem for designing, conducting, and analyzing experiments for an annual fee of $1000 to $2800. The Emotiv devices have been compared to research-grade EEG systems for recording auditory event-related potentials (Emotiv EPOC) [34]. Moreover, modified versions of these devices have been employed in research on brain-mobile interaction, the development of a portable real-time neuroimaging system, and the detection of epileptiform abnormalities [[35], [36], [37]]. On the other hand, DreamMachine differs most significantly from the Emotiv product range in terms of price and its commitment to supporting open science. DreamMachine hardware features enhance accessibility, enabling more people to conduct research and utilize the device. The source code for both the devices and the app, including alternative versions, has been published to facilitate easy adaptation for different research goals.

In conclusion, DreamMachine is valuable to existing mobile EEG devices such as Emotiv, SMARTING mobi, and the device presented in Bateson’s study [38]. The emphasis on low cost, open source, open hardware, and the easy adaptation of the companion app sets DreamMachine apart. Its performance is comparable to other state-of-the-art devices while it is available at a fraction of their cost.

The following five points demonstrate how the DreamMachine hardware can be utilized to support both traditional and innovative laboratory tasks:

• **Affordable High-Quality EEG Data Acquisition**: The DreamMachine provides an open-source, cost-effective solution for collecting 24-channel EEG data, enabling researchers to conduct experiments without the high financial barriers associated with commercial systems.
• **Mobile and Flexible Experimental Designs**: Its lightweight, portable design with Bluetooth connectivity allows researchers to perform EEG recordings in diverse environments, including outside traditional laboratory settings, for studies requiring mobility or ecological validity.
• **Customizable Software for Diverse Applications**: The companion Android application, EEGDroid, is designed to be open-source and highly adaptable, enabling features such as filtering options, data formats, and gain settings to be tailored to experimental needs or integrated with other tools.
• **Support for Multimodal Research**: The system's ability to capture additional signals like EOG and ECG enables multimodal studies, making it ideal for research in neurofeedback, brain-computer interfaces, and psychophysiology.
• **Enhancement of Educational and Training Programs**: Its open-source nature and low cost make the DreamMachine an excellent tool for educational purposes, allowing students and early-career researchers to learn EEG techniques and experiment with hardware development in a hands-on manner.

## 3 Design files summary

All aspects of the project design were developed using freely accessible software tools to ensure broad accessibility and reproducibility. The circuit diagrams for the hardware were created with the EAGLE program (autodesk.com/products/eagle), a versatile and widely recognized schematic editor for electronic design. The companion application, EEGDroid, was programmed in the Java programming language to enhance compatibility with Android devices, enabling real-time data acquisition, visualization, and hardware configuration. MATLAB was utilized for further data analysis, allowing for robust signal processing and statistical evaluation of the EEG recordings. Additionally, the complete project files, including hardware schematics, firmware code, and application source code, have been made available in an open-access repository, facilitating customization, refinement, and adaptation for diverse research applications.

The provided files were designed using an open-source tool for hardware development. Notably, the entire development process, including both software and hardware integration was completed using freely accessible software under an open-source license like C, MATLAB, and Java programming languages. In the case of the EEG caps and electrodes, there is no requirement to build additional hardware components, as the system has been designed to integrate seamlessly with existing setups.

Table 2 presents the PCB design, which includes schematic (.sch), Board Layout (.brd), Gerber (.zip), and Bill of Materials (BoM) files for constructing the hardware. The firmware code, which is developed in C programming language, operates the device’s core functionalities, and the app source code, which is in Java programming language, handles data processing and configuration.

**Table 2:** Design file summary.

**Design file name**	**File type**	**Open-source license**	**Location of the file**
PCB design	*.brd* *.sch* *.pdf* *.csv* *.zip*	*GNU General Public License v3.0*	https://doi.org/10.5281/zenodo.14627406 *https://github.com/paria-samimi/dream-machine-eeg/tree/main/Board%20Design*
Firmware code	The source code is in the C programming Language	*GNU General Public License v3.0*	https://doi.org/10.5281/zenodo.14627406 https://github.com/paria-samimi/Traumschreiber/tree/master/Traumschreiber_BLE_Code
App Source code	The source code is in Java Programming Language	*GNU General Public License v3.0*	https://doi.org/10.5281/zenodo.14627406 https://github.com/denizmgun/EEG-Droid/tree/d72ffb9ea78de1f27fe0690aa12f09323f0b82c2

## 4 Bill of materials

As previously noted, detailed electronic files and Android application resources are available in the Github and Zenodo repositories. All data are distributed under the GNU General Public License Version 3 (GPLv3), ensuring open-source accessibility.

While Table 3 provides partial information about key components, the complete design details can be accessed at https://doi.org/10.5281/zenodo.14627406, in the Board Design section.

**Table 3:** Bill of materials summery.

**Designator**	**Component**	**Number**	**Cost per unit (Euro)**	**Total cost −** **(Euro)**	**Source of materials**	**Material type**
ADC1-ADC3 (Analog to Digital)	AD7779ACPZ ADC Chip	3	12.00	36.00	Link	Semiconductor
J1 (Header)	SHF-105–01-L-D-SM-TR	1	2.50	2.50	Link	Metal
BC832 (Bluetooth)	BC832	1	10.2	10.2	Link	Other
CLOCK	DSC1001DI5-008.1920 T	1	1.8	1.8	Link	Semiconductor
CHARGER	BQ24072TRGTR	1	1.8	1.8	Link	Semiconductor
RESET	TL3780AF330QG	1	0.26	0.26	Link	Semiconductor
ESD1-ESD7 (ESD protection diodes)	TPD4E1B06DRLR	7	0.4	2.8	Link	Semiconductor
Micro USB Type B Connectors	2040002–1	1	1.8	1.8	Link	Other
Battery Holder	1051	1	1.7	1.7	Link	Plastic

**Additional Information:**

1. The PCB for the project was designed using a standard electronic design tool and manufactured by a European company.
2. All components were carefully chosen to ensure compatibility with the DreamMachine design and were sourced from reputable suppliers.
3. The listed prices in the Table 3 reflect component costs at the time the DreamMachine was assembled in 2021. Current prices can be found by following the provided links for each component.
4. Costs related to PCB manufacturing and surface-mount technology (SMT) assembly are not included in the total.
5. Additionally, suitable scalp electrodes are required for use with the system. These can be selected based on the user’s preferences and the specific requirements of their experimental design.

## 5 Build instructions

The DreamMachine hardware system is built through a series of well-defined steps, including PCB design and assembly, firmware programming, and application installation. The process begins with the fabrication of the PCB using Gerber files provided in the project's open-source repository. After the PCB is manufactured, the necessary electronic components are sourced, as specified in the BoM. These components are carefully mounted onto the PCB, employing reflow soldering for surface-mount devices (SMDs) and manual soldering for through-hole components. The assembled PCB seamlessly integrates the analog, digital, and power supply sections, forming the foundation of the hardware system. A 3.7 V, 650 mAh lithium-polymer battery is then connected to the power supply circuit, providing up to six hours of continuous operation on a single charge.

**Fig. 5:**
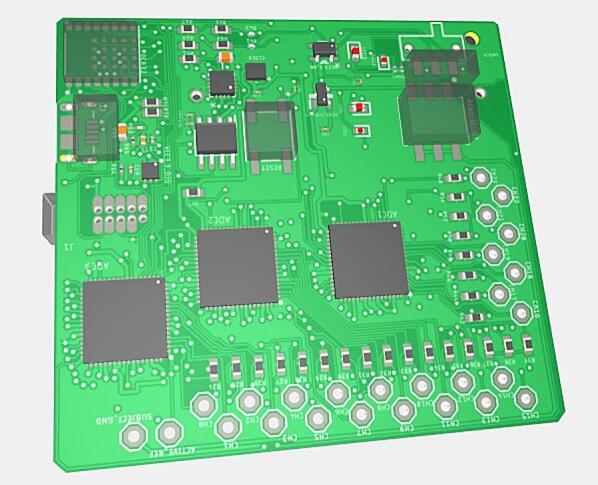

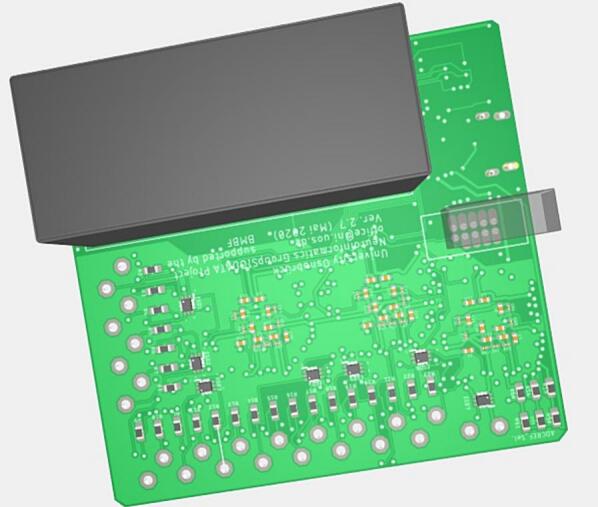
3D view of the DreamMachine PCB. The left image displays the top side of the PCB, while the right image shows the bottom side, highlighting the assembled components and circuit layout.

Fig. 5 provide detailed visualizations of the assembled DreamMachine PCB from both the front and bottom perspectives, highlighting the layout and arrangement of its components.

The right image shows the bottom side of the PCB, which prominently features the routing of copper traces connecting the various electronic components. This side also includes the soldering points for surface-mount and through-hole components, ensuring stable electrical connections. The battery placement is visible, showcasing how it integrates seamlessly into the overall design for compactness and efficient power management. The left image presents the front side of the PCB, which houses the core functional components of the DreamMachine system. It includes three ADCs, each responsible for processing EEG signals from multiple channels, alongside the BLE transmitter module for wireless data communication. The arrangement of these components reflects a thoughtful design optimized for minimizing signal interference and ensuring efficient data flow. Together, these 3D images provide a clear representation of the DreamMachine’s hardware architecture, illustrating the integration of its analog, digital, and power management sections.

Once the DreamMachine PCB board is designed and manufactured, a multimeter is used to test all connections to ensure reliability. This step confirms the integrity of the connections and verifies that no short circuits are present before proceeding further. The next step is programming the firmware into the system. The firmware files, available in the project's repository, enable the system to interface with the ADCs using Serial Peripheral Interface (SPI) protocols and to transmit EEG data via BLE. The microcontroller is flashed with the firmware, process the data through filtering and downsampling techniques, and encode the results into packets for seamless BLE transmission. Once the firmware is installed, debugging tools are employed to verify the functionality of the data pipeline and ensure successful communication with paired devices. For more detailed information on how the firmware configures the ADCs and manages data processing, refer to the methodology described in the previous publication [16].

The final step is installing the EEGDroid application on an Android device. This application, available for download from the project's repository. Once both the hardware and software components are fully set up, electrodes are connected to the DreamMachine system to enable EEG signal acquisition. The system's performance is then tested by recording sample EEG signals and visually inspecting them using the application's real-time monitoring features to ensure signal integrity and proper functionality. During operation, standard safety guidelines for EEG recording are followed, including proper skin preparation for electrode placement and securing the battery connections to prevent short circuits or overheating.

It is important to emphasize that the detailed PCB design, firmware code scripts, and the EEGDroid application are provided in the project's repositories (GitHub). Comprehensive build instructions for both the hardware and firmware are thoroughly outlined in our previous publication [16].

## 6 Operation instructions

**Fig. 6:**
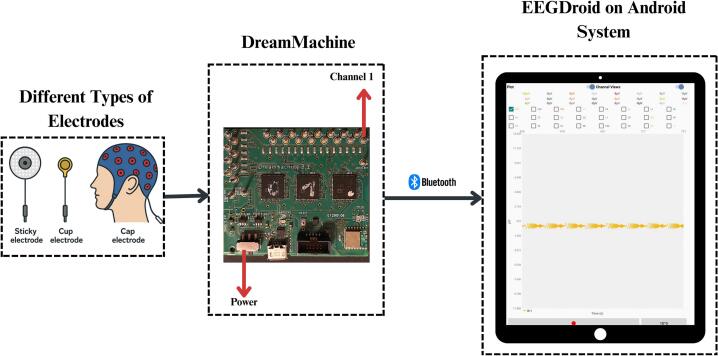
The procedure begins by attaching the selected electrodes—such as sticky, cup, cap, or other types—to the subject’s scalp. These electrodes are then connected to the corresponding input pins on the DreamMachine. Once connected, the DreamMachine is paired with the EEGDroid application via Bluetooth. After choosing the appropriate configuration within the app, brain activity recording can begin. The recorded signals are saved as a CSV file, allowing researchers to conduct further data analysis.

Fig. 6 presents the general workflow for conducting an EEG experiment using the DreamMachine system. The procedure begins with selecting and attaching EEG electrodes to the subject’s scalp. Users can choose from a variety of electrode types, depending on the needs of the study and available equipment. Electrodes may be placed using a standardized cap or manually positioned according to the 10–20 system. The DreamMachine supports up to 24 channels, although users are not required to utilize all of them. The system is designed for complete flexibility, allowing researchers to select only the number and locations of channels that are relevant to their specific experimental design. Reference and ground electrodes are typically placed on the mastoid bones behind the ears, although this can be adjusted to meet the standards of the research protocol.

The other ends of the electrodes are securely connected to the DreamMachine’s 26-pin input interface. Once all electrodes are in place, the device is powered on. The EEGDroid companion app should be installed on an Android device in advance of use. After turning on the DreamMachine, the app detects the device via Bluetooth and enables a seamless pairing process. Users can then configure device parameters in the app, including sampling rate, resolution, gain, filters, and other settings. Although a recommended default configuration is available, all settings are fully user-adjustable to accommodate diverse experimental needs.

**Fig. 7:**
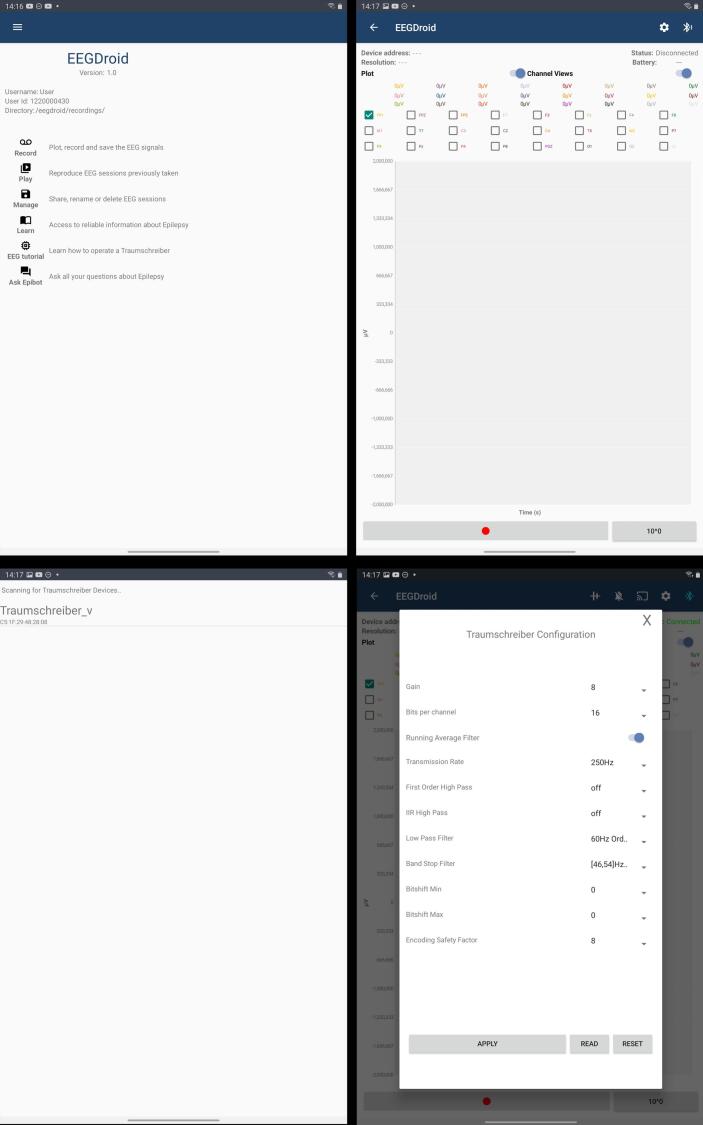

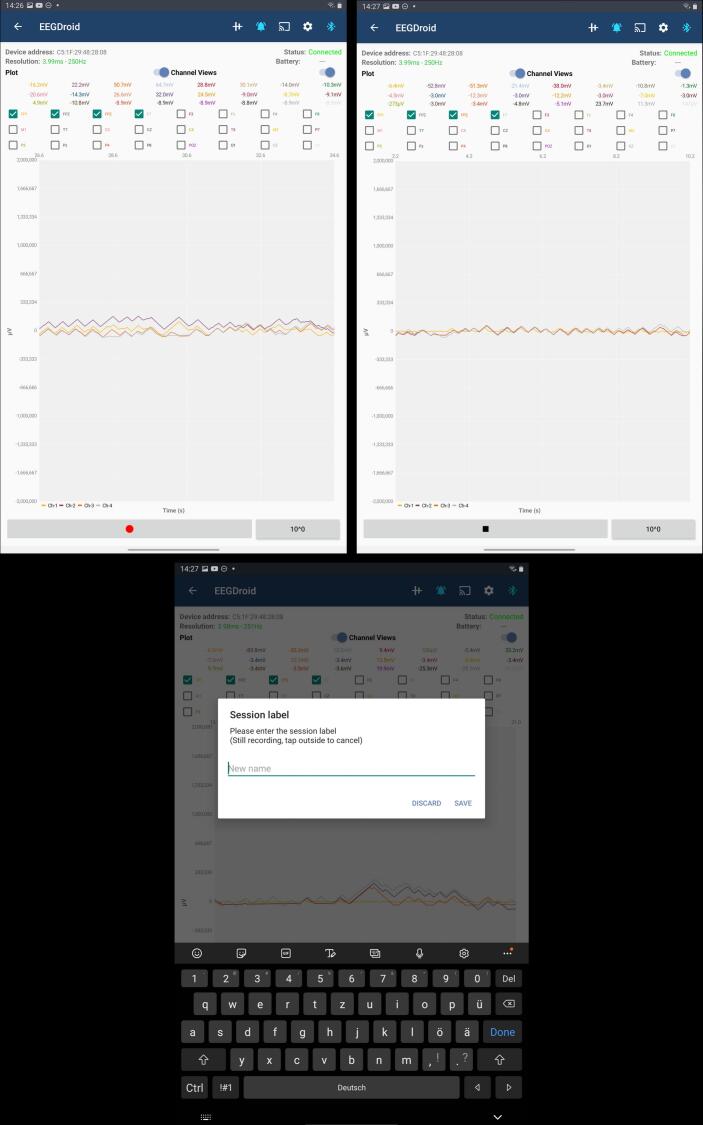
The prototype of the EEGDroid.

In Fig. 7, the prototype of the EEGDroid application is presented. The prototype Android application is designed to record EEG signals on Android devices. The process of using, connecting, and configuring the application is demonstrated through figures. The first menu of the EEGDroid application is accessible at the top left corner, where the “Record” button can be selected to initiate the recording process. Upon selecting “Record,” the display menu is shown. To connect the DreamMachine hardware to the application, the Bluetooth symbol on the top right must be selected. When the DreamMachine hardware system is powered on, the name “Traumschreiber_v” appears in the list of detected Bluetooth devices. Once selected, the “Gear” icon, located next to the Bluetooth icon, allows configuration of the application to optimize EEG signal quality. Configuration options are displayed in the accompanying images, with further details explained in subsequent sections.

After the appropriate configuration is selected, EEG signals can be visualized on the screen by selecting the “Bell” symbol located at the top right. All 24 channels are displayed in distinct colors, and individual channels can be clicked to assess signal quality and behavior. The recording process begins by selecting the red button at the bottom left. Upon completion of the experiment, recording can be stopped, and recordings can be labeled as needed. The application provides a summary of the recording’s performance and saves the data in “CSV” format for further analysis.

## 7 Validation and characterization

With the increasing availability of low-cost and mobile EEG systems, a growing number of studies have assessed their signal quality in comparison to research-grade equipment [[47], [48], [49]]. For instance, Melnik et al. (2017) examined variability across different EEG systems relative to the variability observed between subjects and across repeated sessions, using the same experimental paradigm applied to four different devices [47]. This type of comparative validation helps clarify how system-related factors influence EEG data quality. Building on this approach, the current study employed the well-established eyes-open/eyes-closed paradigm to evaluate the DreamMachine's performance relative to a standard clinical-grade EEG system. This task is frequently used in validation studies due to the robust and predictable increase in alpha-band power observed during eye closure—a neural marker widely supported in the literature [48,49].

The validation was conducted at the EEG laboratory of Osnabrück University, where the DreamMachine was tested alongside the asalab™ system from ANT Neuro. The same participants completed the eyes-open and eyes-closed task with both systems under matched experimental conditions. The resulting alpha-band differences between eyes-open and eyes-closed conditions were analyzed to assess the DreamMachine’s ability to detect established neural patterns and manage baseline noise levels effectively. These findings provide preliminary evidence that the DreamMachine can produce reliable EEG recordings suitable for cognitive paradigms, aligning with earlier demonstrations of the capabilities of other mobile EEG platforms [48,49].

Ten subjects, five males and five females aged between 18 and 25 years with normal or corrected vision, participated in the study. According to previous studies, a sample size of 10 subjects is commonly selected for experiments involving eyes-open and eyes-closed conditions, as it has been shown to be sufficient for detecting meaningful differences in neural activity. These studies suggest that, given the controlled nature of the experiment and the focus on individual brain responses, a sample size of 10 provides reliable and valid results without the need for larger groups [47,48]. Before commencing the experiment, participants were not subjected to neurological, chronic, or psychological evaluations. They were requested to disclose any such conditions if applicable. None of the participants reported any disabilities or the aforementioned medical issues to their knowledge. Furthermore, participants were instructed to abstain from consuming beverages containing alcohol or caffeine for at least three hours before the commencement of the experiment. For a more accurate comparison, the same cap (the waveguard™original ANT Neuro EEG) was used, and the experiment was conducted in the same location, with the same subjects, and under the same conditions. The experiment involved participants closing and opening their eyes for two-minute intervals, repeated five times. This resulted in one part of the experiment lasting 20 min, and the entire experiment taking 40 min for each subject. In this study, the experiment commenced with the eyes-closed condition. The used cap, allowed for the continuous recording of signals from 24 electrodes, which were positioned by the international 10–20 system.

Similar research on DreamMachine and standard EEG devices for a single subject has been previously conducted and documented [16]. The primary objective of the current study was to ensure the comparability between the DreamMachine and a conventional EEG device across a larger number of subjects, thereby demonstrating the reliability of the DreamMachine in a more extensive sample.

### 7.1 Methodology

**Fig. 8:**
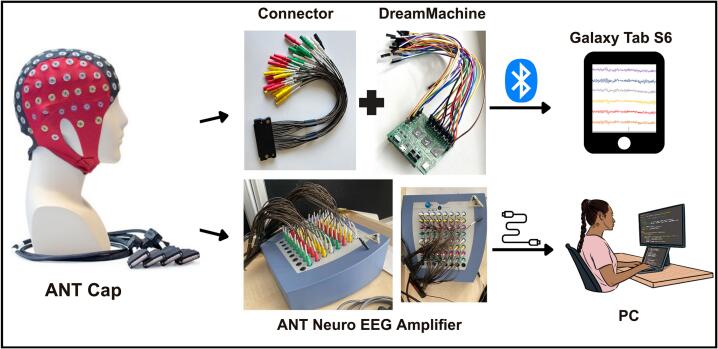
The ANT cap is first connected to the ANT Neuro EEG amplifier, which is linked to the ASALAB software system on a PC for standard EEG recording. In a separate setup, the same ANT cap is connected to the DreamMachine using a connector. In this configuration, EEG signals are transmitted via Bluetooth from the DreamMachine to the EEGDroid application installed on a Galaxy Tab S6 tablet. The ANT cap image is adapted from ANT Neuro [53].

Each subject was seated on a chair at a distance of 90 cm from the monitor, with the experiment receiving approval from the Osnabrueck University Ethics Committee for the Protection of Human Subjects. 24 channels of the waveguard™ original ANT Neuro EEG cap are connected to the subjects, and for all subjects, the left earlobe was used as a reference point, while the right earlobe served as the ground. In the initial phase of the experiment, recordings were made using the Standard EEG system. The cap was affixed and connected to an amplifier system, facilitating signal transmission to a computer placed in the laboratory. Fig. 8 illustrates the setup of the experiment. Subsequently, the recorded data was processed and stored using the ASALAB analysis software and data acquisition occurred at a sampling rate of 1024 Hz. Once all necessary steps were completed, the impedance of all 24 channels was checked using ASALAB software to ensure it remained below 5kΩ. Upon ensuring all channels were correctly connected, the subject was instructed to look left and right and blink to verify dissimilar behavior in all channels, confirming the proper connection of the reference and ground. Next, the subject was trained on the experiment protocol. The experiment was initiated after confirming that all equipment was properly connected and functioning. The standard EEG system was used first for all subjects, followed by the DreamMachine.

In the case of DreamMachine, it was wirelessly linked via Bluetooth to a tablet (Samsung Galaxy Tab S6 Lite, 64 GB) running the EEGDroid application, facilitating the recording and storage of the EEG signal. The default recording settings in the EEGDroid application included a notch filter with a frequency range from 46 Hz to 54 Hz (fourth-order filter), a gain level of 8, and a low-pass filter set at 60 Hz (sixth-order filter) was used for this experiment. Additionally, data acquisition was performed at a sampling rate of 250 Hz, with the LSL-recorder application [50], which was used to record and save the data as an xdf file. Triggers were systematically documented in both phases of the experiment to denote instances of participants closing or opening their eyes. In the eyes-open condition, participants were required to direct their gaze toward a fixation cross displayed on the monitor, and in both conditions, they were asked not to move their body posture.

### 7.2 Data extraction

The recorded data were obtained in two different formats due to the use of different devices. The standard EEG system produced data in the .cnt format, while the DreamMachine generated data in the .xdf format. Initially, the data were imported into MATLAB using Fieldtrip in two separate files, after which the same script was used for their analysis. First, a visual comparison was made between the plots generated by the DreamMachine system and those produced by the standard EEG system in time domain. This involved scrutinizing the plots from the DreamMachine system to identify prominent features under two different conditions (eyes-open, eyes-closed), such as eye movements, eye blinks, Alpha waves, and Delta waves. Subsequently, the same analysis was conducted on the standard EEG system’s plots. Following this, the data were denoised and analyzed in both time and frequency domains.

During the recording process, the EEGDroid application filtered out the alternating current (AC) to clean the data and eliminate noise, so this step was omitted from subsequent preprocessing steps. In the next stage, after excluding noisy channels, the signal was segmented into 40 sections, resulting in 30-second epochs for both eyes-open and eyes-closed conditions. Following this, the power spectrum for each epoch was calculated using the Fast Fourier Transform (FFT). Then, the same statistical analysis was applied to both devices. To address the multiple comparison problem (MCP) associated with multidimensional data, a rigorous cluster-based permutation test was employed. This test aimed to determine the significance probability (p-value) for multiple time points by computing clusters, rather than conducting individual statistical tests for each time point. Essentially, clusters were computed based on t-values, then their permutation was determined, and the maximum values were used to calculate the significance probability. For both datasets, the t-value was derived from the independent samples t-test, with an alpha threshold set at 0.01 for both conditions. Additionally, the Monte Carlo method was used to calculate the significance probability of the clusters. The results of the cluster-based permutation test were then presented in a topographical plot, illustrating power spectra for both conditions. The script for analyzing the experiment is available on a GitHub repository.

In summary, an analysis was conducted on each plot generated by the DreamMachine system to identify the presence of the Alpha rhythm in the occipital head channels during the eyes-closed condition, as well as detecting eye blinking, eye movements, and Delta rhythm during the eyes-open condition. This process was then repeated for the plots generated by the clinical EEG system. It was subsequently determined whether the two systems exhibited analogous components in each corresponding epoch or if specific features were unique to either of the recordings.

### 7.3 Data analysis

The preprocessing steps were completed after the data were divided into different conditions. The data were then plotted in the time domain to compare the signals from both EEG systems. The signals from all subjects, recorded with both systems, were plotted and visually inspected. The behavior of the signals in both eyes-open and eyes-closed conditions was similar across both systems.

For data to be properly analyzed in the frequency domain, all cleaned data sets were analyzed using the multi-taper method with Fast Fourier Transform (FFT) to compute power spectra. To achieve better results, the data were examined in various frequency bands to gain insights into brain activity behavior under different conditions. The selected frequency bands in this project are Delta (1–4 Hz), Theta (4–8 Hz), Alpha (8–12 Hz), and Beta (12–30 Hz). Finally, a cluster-based permutation test was employed to compare the power spectral density between the ‘open’ and ‘closed’ conditions across the 24 channels [16]. As a result, seven channels, namely FP1, FP2, FPz, POz, O1, O2, and OZ, are selected as the most effective channels. The results of the analysis are presented in the next section.

### 7.4 Results

**Fig. 9:**
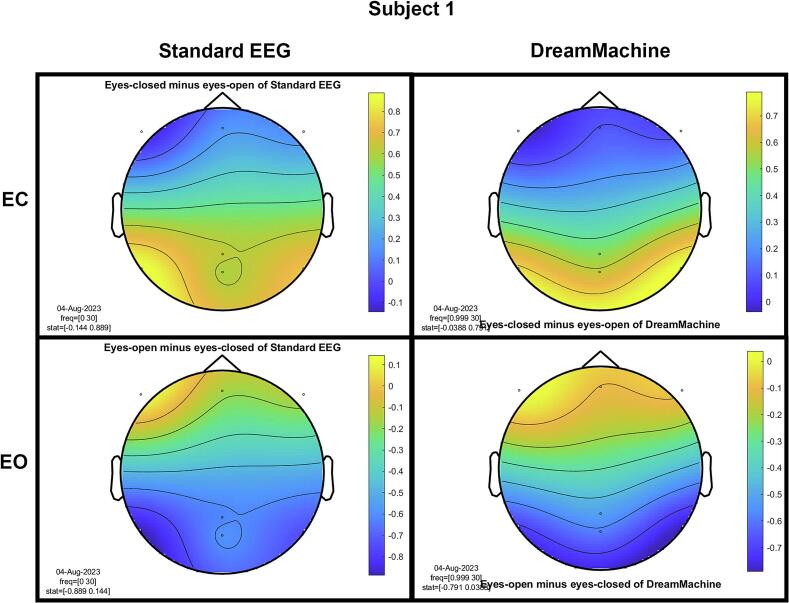

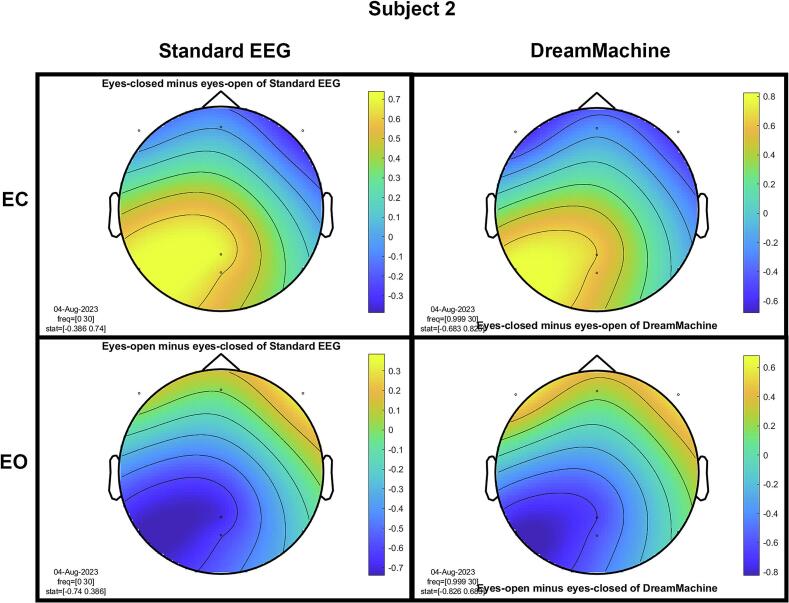

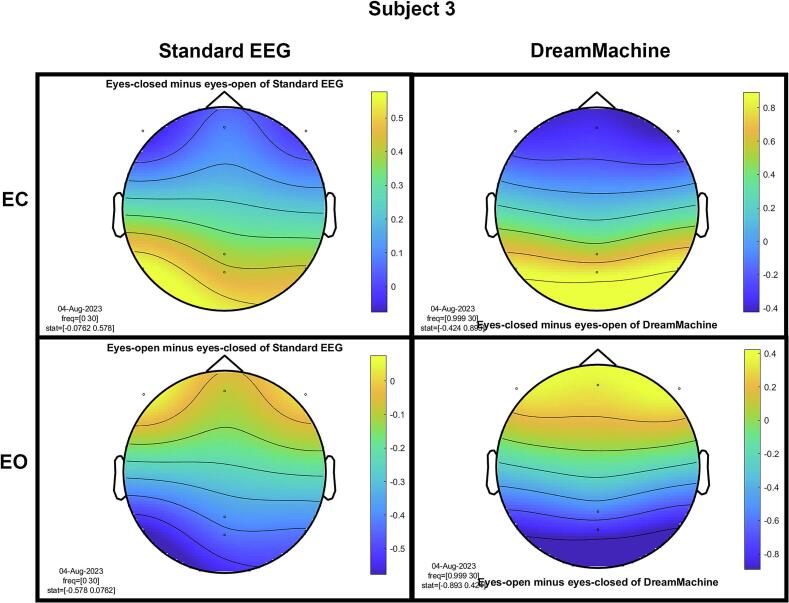

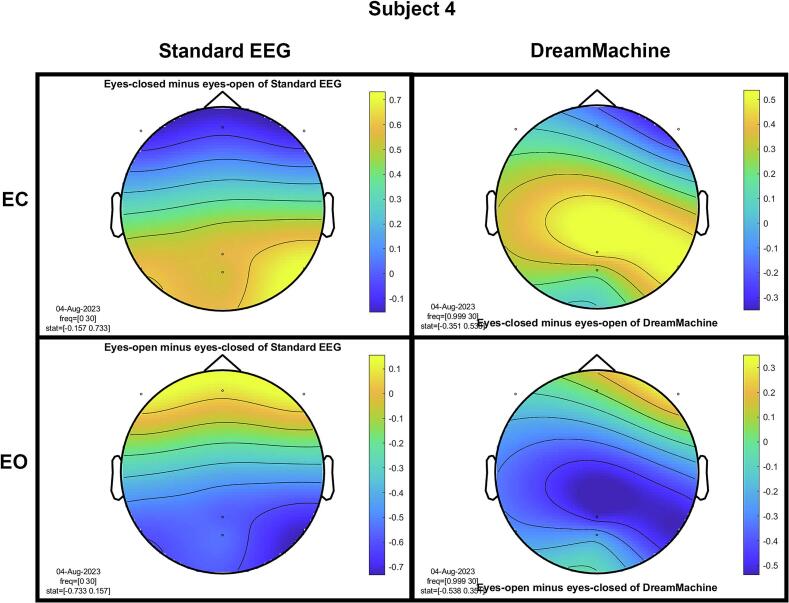

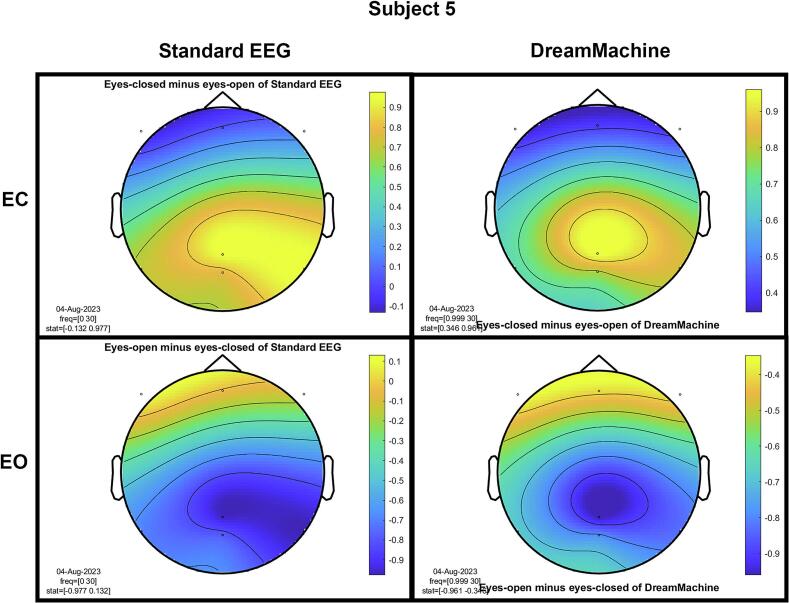

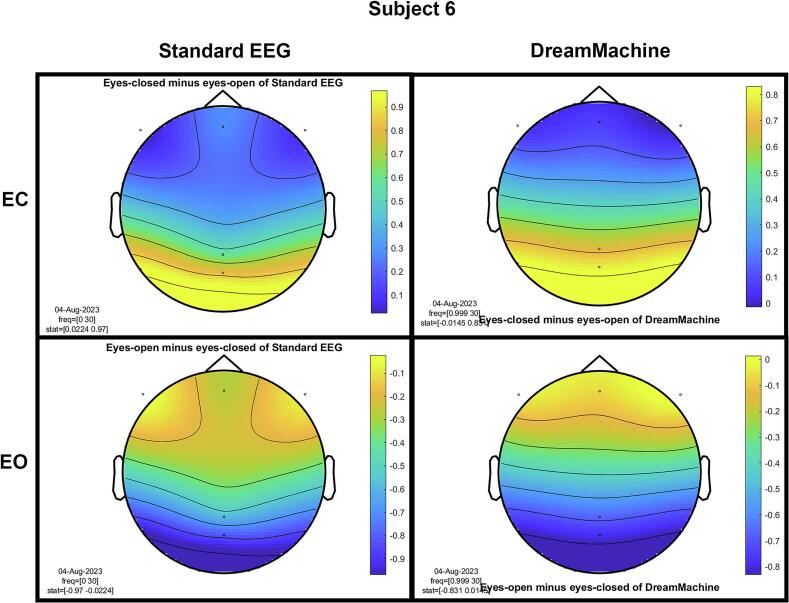

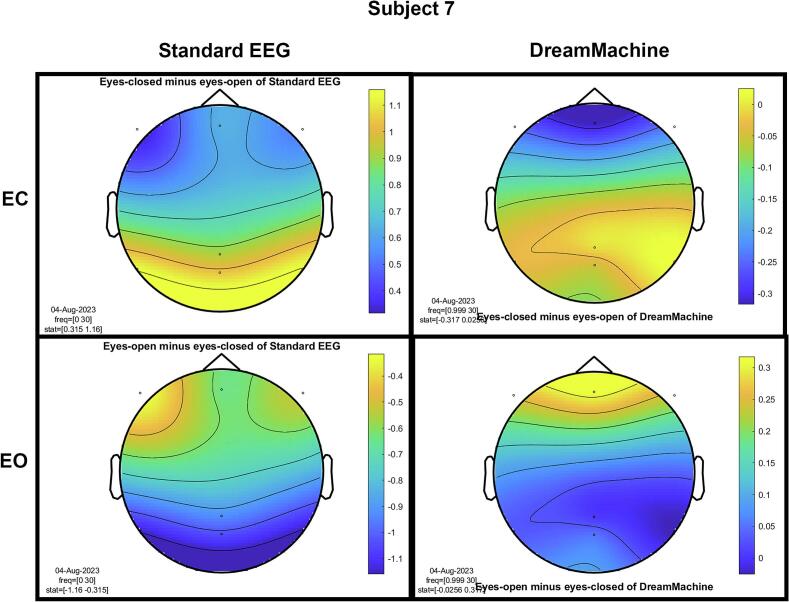

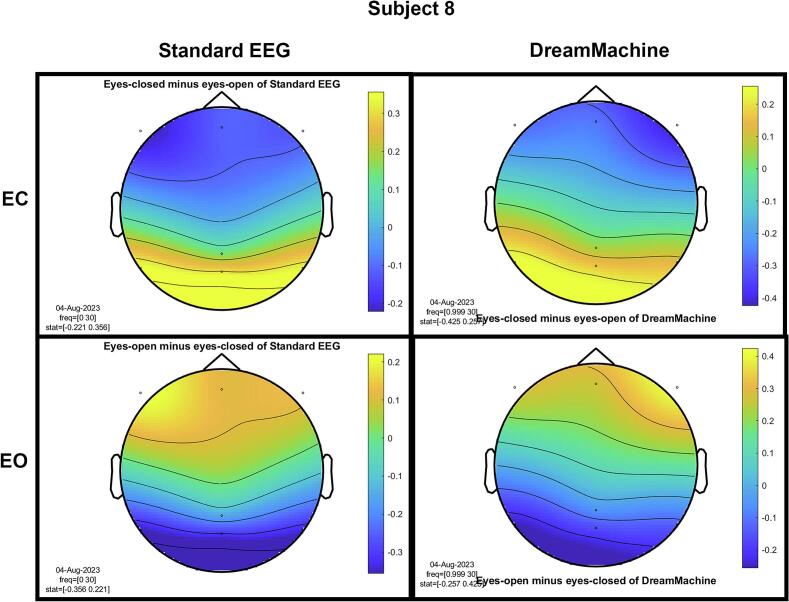
The results from the cluster-based permutation test on the topographical power spectra are illustrated for every participant. These results are categorized by condition and EEG system for each individual. The topographies on the left side of each figure are from the standard EEG system, while those on the right side are from the DreamMachine system. The top two topographies also correspond to the eyes closed condition, and the bottom two topographies represent the eyes open condition.

The results of the cluster-based permutation test for eight participants are shown in Fig. 9. It is evident that similar brain activity signals were captured by both EEG devices. In the eyes-closed condition, a significant increase in activity (around 10 Hz) was observed in the occipital region for all participants. This finding highlights the prevalence of alpha activity during resting states. Additionally, increased activity in the frontal region was evident in the eyes-open condition, corresponding to heightened visual activity during eyelid movement and information processing across the entire cortex. Despite some noise being present in the signals from both devices, their consistency across conditions is apparent.

**Fig. 10:**
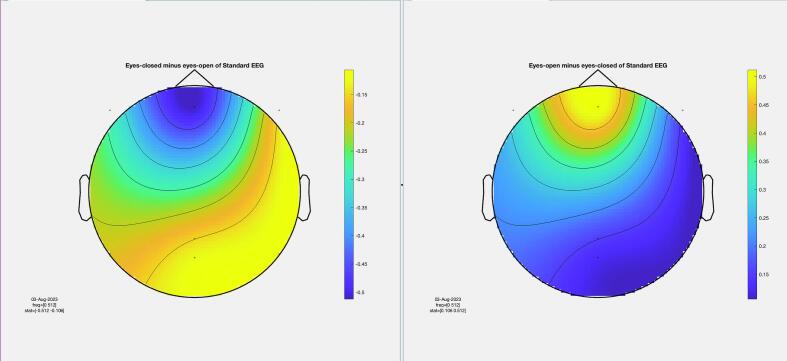

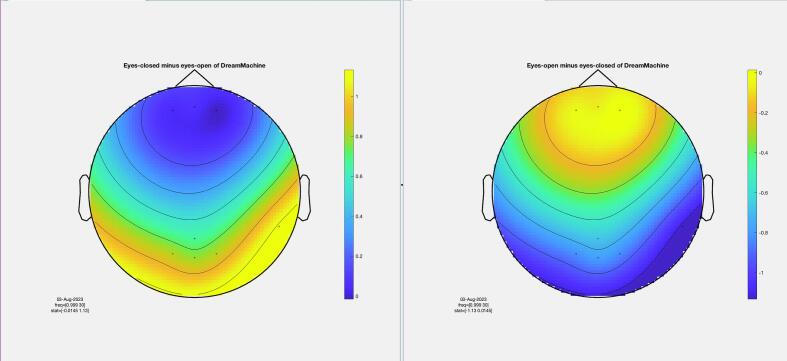
An extensive statistical analysis was performed using the cluster-based permutation test. The right side displays the results for “eyes open minus eyes closed,” while the left side shows the results for “eyes closed minus eyes open.” Panel A presents data from the Standard EEG system, whereas Panel B highlights the results from the DreamMachine system.

Fig. 10 showcases topographical plots that visually represent statistically significant differences in power spectral density between the “eyes closed minus eyes open” and “eyes open minus eyes closed” conditions in both EEG systems. Distinct color-coded regions highlight clusters of electrodes with significant differences, with more intense colors signifying lower p-values (p < 0.01). These findings indicate that higher power spectral density is predominantly observed in the occipital region. It is also highlighted that the same behavior is observed in both EEG systems, which confirms that the results of both EEG systems are roughly the same.

**Fig. 11:**
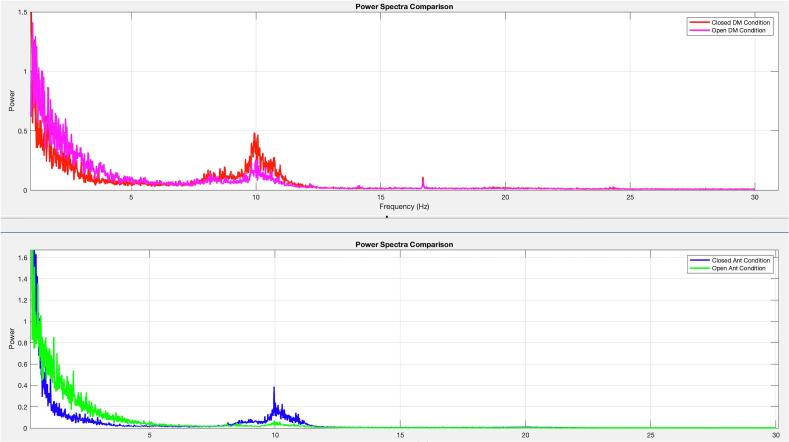
Power spectra diagram of the averaged data for both eyes open and eyes closed conditions and Standard on the top, and DreamMachine EEG systems on the bottom.

The final result is illustrated in Fig. 11 through a power spectra diagram, showing the relationship between power and frequency. For both devices, there is notable power in the range of 0 Hz – 4 Hz (Delta band), particularly in the eyes-open condition. This increased power in the delta band in both standard EEG and DreamMachine systems in eyes-open condition aligns with expected patterns arising from heightened cortical activity during information processing and visual stimulus reception. Additionally, a significant peak in power is observed around 10 Hz for the eyes-closed condition, attributed to pronounced alpha activity during rest, consistent with the Berger effect and anticipated results. Overall, both EEG systems produced nearly identical outcomes across both conditions [51].

## 8 Discussion

The DreamMachine was developed as an accessible, open-source, and customizable mobile EEG system that balances affordability with functional performance for a wide range of EEG applications. Its primary goal is to serve as a flexible platform for educational use, exploratory research, and rapid prototyping in neuroscience and neurotechnology. One of its key strengths is its open design; all hardware and software components are fully documented and modifiable, allowing users to explore system architecture, tailor the platform to their specific needs, and contribute to its ongoing development. Its low cost further enhances its accessibility, making it particularly well suited for classroom environments, workshops, hackathons, and research projects in resource-limited settings.

The potential of the DreamMachine system for cognitive studies is supported by the experimental results presented in this paper. In the eyes-open/eyes-closed paradigm, both the DreamMachine and a standard mobile EEG system consistently demonstrated the expected increase in alpha-band power during eye closure, a well-established indicator of neural activity. This consistency across systems confirms that the DreamMachine can reliably capture key EEG features. While it is not intended to match the performance of high-end systems, these results suggest that DreamMachine is well suited for cognitive neuroscience applications, particularly in contexts where cost or accessibility are limiting factors.

One notable limitation of the DreamMachine system is the absence of an impedance checking feature. While the companion app provides a live view of each channel’s signal for visual inspection, this method is subjective and may be insufficient for detecting poor electrode contact—especially for inexperienced users. Without automated impedance feedback, issues such as high electrode impedance or poor connections may go unnoticed, potentially degrading data quality and affecting the accuracy of EEG analysis. This limitation is particularly relevant in field or educational settings, where users may lack the expertise to identify and resolve such problems in real time. Another constraint relates to the number of channels. Although DreamMachine offers 24 EEG channels, which is sufficient for many standard experiments, it may not provide the spatial resolution required for advanced source localization or ICA-based artifact rejection that benefits from higher channel density.

Future work will include formal comparisons between the DreamMachine and other mobile EEG systems across a wider range of experimental tasks. In parallel, efforts are underway to develop enhanced software tools for artifact rejection and signal interpretation, as well as to explore integration with wearable and IoT-based technologies. In addition, comparative tests have already been conducted between the DreamMachine and other systems—such as the Somno HD Eco [52]—in the context of sleep-related studies involving nightmares and sleep disorders. The results will be detailed in a forthcoming publication. Finally, the DreamMachine’s potential for ambulatory monitoring and use in ecologically valid, real-world settings will be further investigated through long-term studies.

**Ethics statement**

The DreamMachine is a device that resembles a standard medical device and is intended solely for educational and experimental use, developed with adherence to biomedical safety standards. The participants in this study were researchers from electronic and cognitive science fields. They were fully informed about the device's purpose and the procedures involved, and written informed consent was obtained before participation.

The volunteers were healthy individuals with no medical history of epilepsy or other conditions that could impact the experimental procedures, and no scars were present in the experiment. The research was conducted in compliance with the Declaration of Helsinki and approved by the Ethics Committee of Osnabrück University. This ensures the study aligns with ethical standards for research involving human subjects.

## CRediT authorship contribution statement

**Paria Samimisabet:** Writing – review & editing, Writing – original draft, Visualization, Software, Methodology, Data curation, Conceptualization. **Laura Krieger:** Writing – review & editing, Writing – original draft, Software, Methodology, Data curation. **Marc Vidal De Palol:** Writing – review & editing, Software, Resources, Investigation. **Deniz Gün:** Writing – review & editing, Software, Formal analysis. **Gordon Pipa:** Writing – review & editing, Supervision, Project administration, Funding acquisition, Conceptualization.

## Declaration of competing interest

The authors declare that they have no known competing financial interests or personal relationships that could have appeared to influence the work reported in this paper. The first authors are Ph.D. candidates, and all hardware components used in the study were purchased with funding from the SIDDATA project. The funding bodies had no involvement in the design, data collection, analysis, interpretation, manuscript preparation, or the decision to publish the results.
